# Supplementary material for: Diversity and varying predation capacities of culturable Amoebozoae against opportunistic vibrios in contrasting Mediterranean coastal environments
Source: Microbiol Spectr. 2026 Jan 22;14(3):e01138-25. doi: 10.1128/spectrum.01138-25 (PMC12955452; doi:10.1128/spectrum.01138-25)
Supplement: Supplemental material — Fig. S1 to S5; Tables S2, S3, and S6. [file spectrum.01138-25-s0001.pdf]

Supplementary Figures

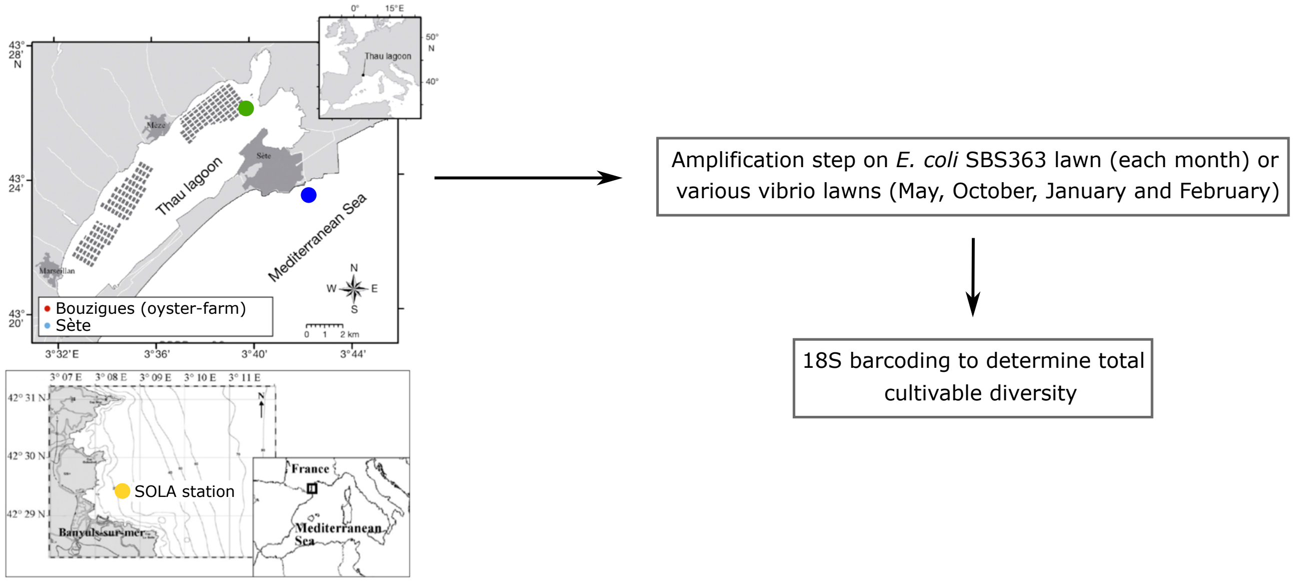

Figure S1. Simplified diagram of the sampling strategy. Briefly, water column and sediment were sampled monthly for one year in the lagoons of Sète, Banyuls-sur-Mer and Thau, near the Bouzigues oyster farming area. Grazers were isolated by selective growth and migration on agar plates coated with E. coli SBS363 lawns. In May, October, January and February, grazer isolation from the same samples was additionally performed on vibrios lawns (V. harveyi A01, V. tasmaniensis LGP32 and V. crassostreae J2-9). All grazers that grew and migrated from the initial sample were recovered and total DNA was extracted for v4-18S barcoding.

A

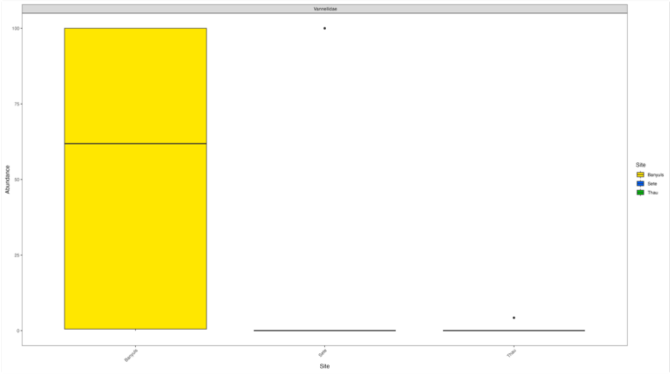

	s.Banyuls	s.Sete	s.Thau	index	stat	p.value
Vannellidae	1	0	0	1	0.735632	0.009

B

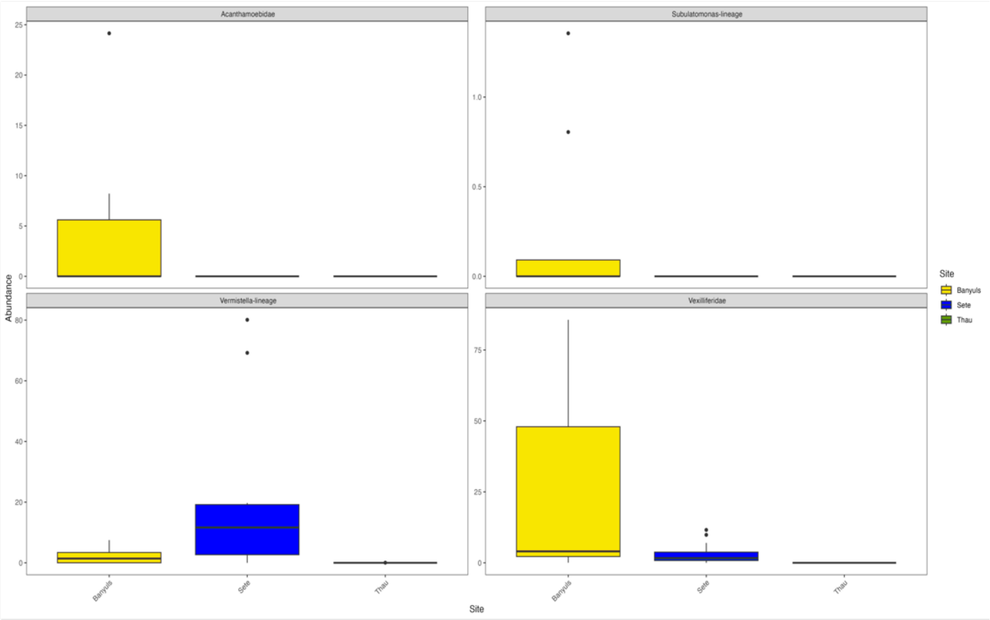

	s.Banyuls	s.Sete	s.Thau	index	stat	p.value
Vexilliferidae	1	0	0	1	0.934671	0.002
Acanthamoebidae	1	0	0	1	0.57735	0.042
Subulatomonas-lineage	1	0	0	1	0.57735	0.042
Vermistella-lineage	0	1	0	2	0.90825	0.001

Figure S2. Indicspecies (Vegan package) analyses on relative abundance data to identify site-specific Amoeboza taxon accoding to the sampling sites in the two different sampling fractions. A. Water samples, B. Sediment samples.

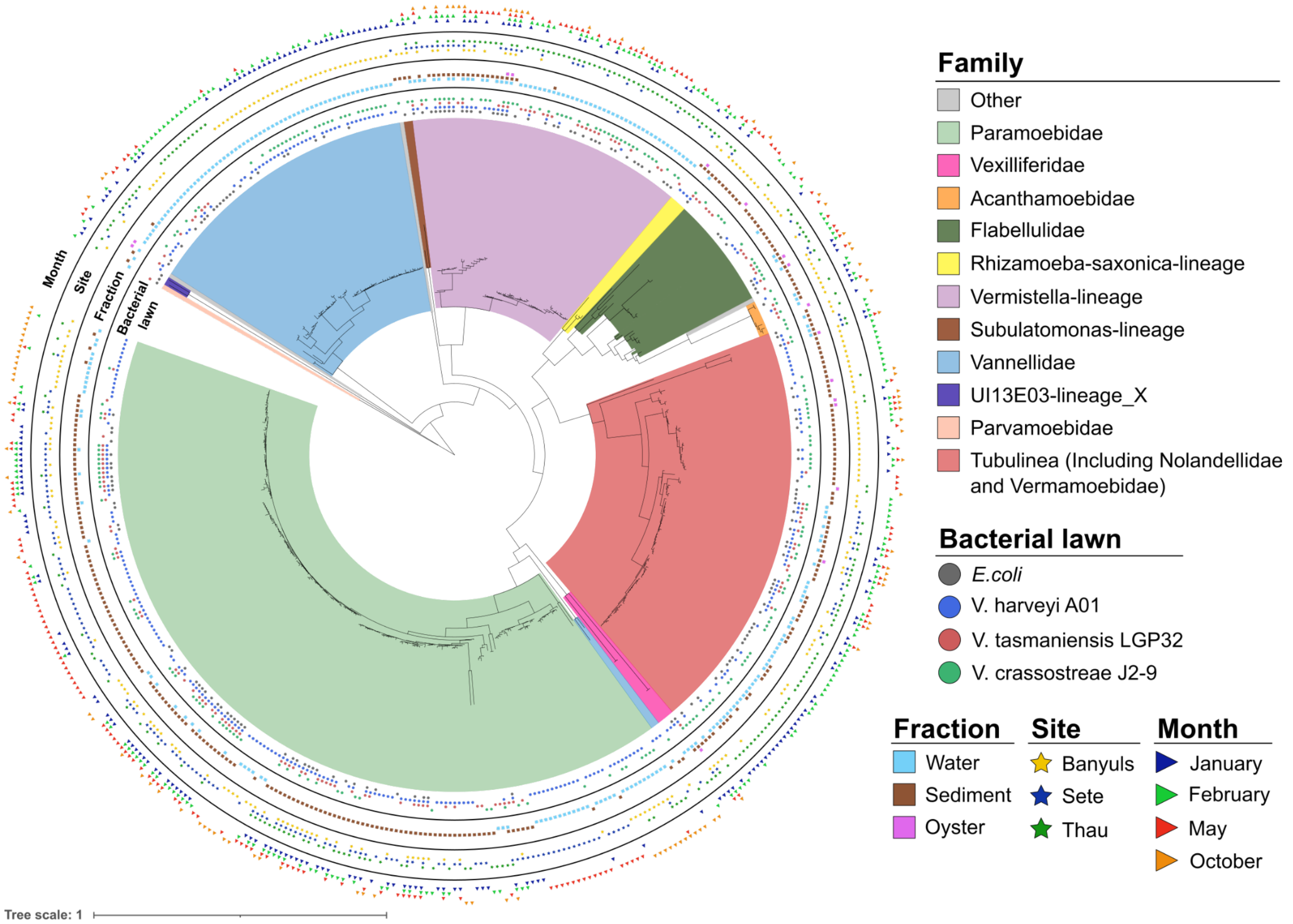

Figure S3. Phylogeny of all ASVs identified on the four different bacterial lawns. The phylogenetic classification of the ASVs was performed using MAFFT and FastTREE FastTree (Maximum Likelihood tree) and annotated using iTOL software, highlighting fraction, location, and season variables.

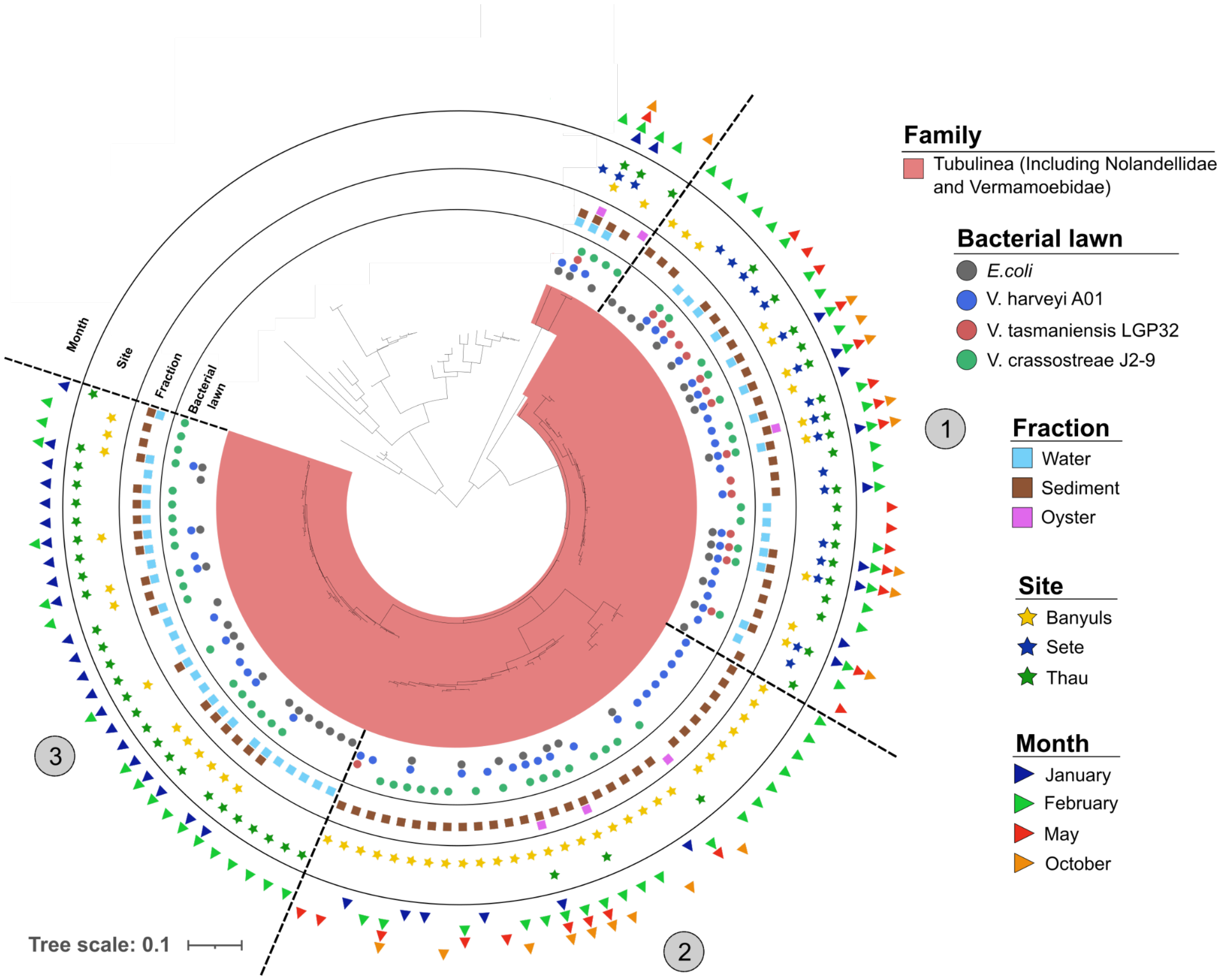

Figure S4. Phylogeny of ASVs highlighting different clades of Tubulinea found in different environments with different predation capacities. The phylogenetic classification of ASVs was performed using MAFFT and FastTREE FastTree (Maximum Likelihood tree) and annotated using iTOL software, highlighting fraction, location and season variables. Some clades appeared to be ubiquitous and mostly generalist as they could grow on the four bacterial lawns like clade 1, whereas clades 2 and 3 appeared to have more restricted habitats with a more limited predation capacity.

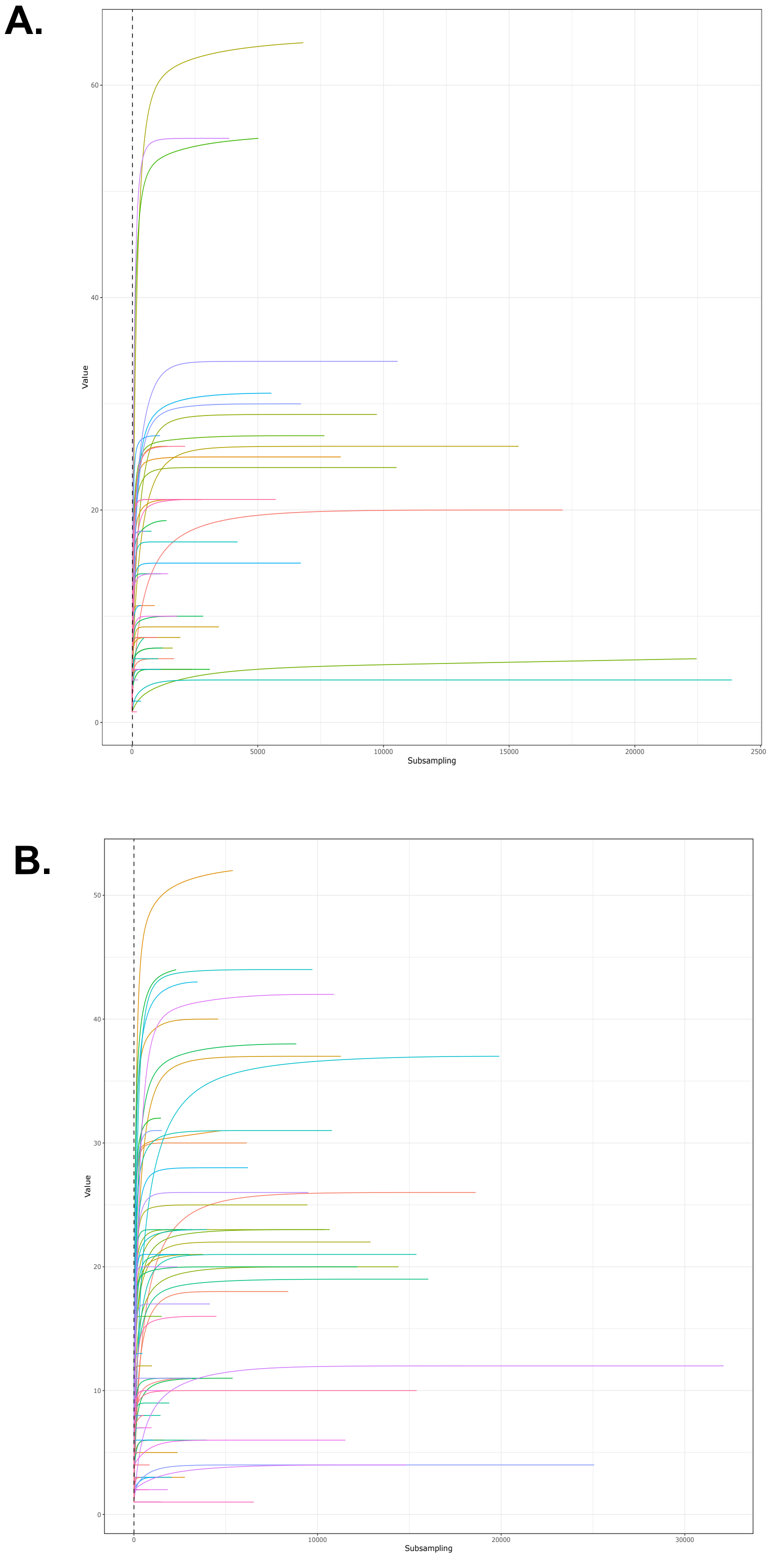

Figure S5. Rarefaction curve analysis. A. Rarefaction curves of sequencing data from the samples cultured on E. coli plates. B. Rarefaction curves of sequencing data from the samples cultured on the three different vibrio plates.

Table S2

(A) Statistics of the alpha diversity using the Chao1, Shannon and InvSimpson diversity indexes on Fraction, Site and Season variables.

ANOVA on Chao1, Shannon and InvSimpson diversity indexes

		Chao1		Shannon		InvSimpson	
		**F value**	**Pr (>F)**	**F value**	**Pr (>F)**	**F value**	**Pr (>F)**
**Fraction**	Water column vs Sediment	6.525	0.0138 (*)	3.734	0.0591	2.349	0.132
**Site**	Banyuls vs Sète	0.698	0.40912	0.002	0.961	0.025	0.875
	Banyuls vs Thau	0.978	0.330	0.010	0.922	0.967	0.333
	Sète vs Thau	0.078	0.782	0.189	0.667	1.061	0.310
**Season**	Winter vs Autumn	1.027	0.32244	1.984	0.174	0.133	0.719
	Winter vs Spring	0.114	0.738	0.113	0.7394	0.466	0.500
	Winter vs Summer	0.383	0.5415	0.199	0.659	0.020	0.890
	Autumn vs Spring	1.432	0.242	3.023	0.0939	0.712	0.406
	Autumn vs Summer	0.551	0.466	0.097	0.75809	0.010	0.921
	Spring vs Summer	0.000	0.990	0.599	0.445	0.231	0.634

(B) Statistics of the alpha diversity using the Chao1, Shannon and InvSimpson diversity indexes Site and Season variables within Water samples.

ANOVA on Chao1, Shannon and InvSimpson diversity indexes

		Chao1		Shannon		InvSimpson	
		**F value**	**Pr (>F)**	**F value**	**Pr (>F)**	**F value**	**Pr (>F)**
**Water**	Banyuls vs Sète	1.09	0.313	0.426	0.523895	0.469	0.504
	Banyuls vs Thau	0.439	0.518	0.483	0.4977	1.562	0.2305
	Sète vs Thau	2.807	0.112	1.413	0.25093	2.589	0.1260

(C) Statistics of the alpha diversity using the Chao1, Shannon and InvSimpson diversity indexes Site and Season variables within Sediment samples.

ANOVA on Chao1, Shannon and InvSimpson diversity indexes

		Chao1		Shannon		InvSimpson	
		**F value**	**Pr (>F)**	**F value**	**Pr (>F)**	**F value**	**Pr (>F)**
**Sediment**	Banyuls vs Sète	1.110	0.306	0.054	0.819	0.000	0.997
	Banyuls vs Thau	1.770	0.205	0.325	0.5775	0.055	0.817
	Sète vs Thau	0.023	0.881	0.122	0.73094	0.054	0.8182

Table S3

(A) Statistics of the beta diversity using the DEseq2 normalization method and Unifrac distance matrix depending on Fraction, Site and Season variables.

Adonis test on multiple variables

Permutation: free

Number of permutations: 9999

		R^2	Pr (>F)
**Fraction**	Water column vs Sediment	0.19817	0.0001 (***)
**Site**	Banyuls vs Sète	0.05936	0.0396 (*)

	Banyuls vs Thau	0.11076	0.0001 (***)
	Sète vs Thau	0.12165	0.0002 (***)
**Season**	Winter vs Autumn	0.04621	0.385
	Winter vs Spring	0.02893	0.5399
	Winter vs Summer	0.02488	0.7264
	Autumn vs Spring	0.04115	0.2992
	Autumn vs Summer	0.04445	0.3417
	Spring vs Summer	0.05616	0.065

(B) Statistics of the beta diversity using the DEseq2 normalization and Unifrac distance matrix depending Site variable within Water samples.

Adonis test on multiple variables
Permutation: free
Number of permutations: 9999

		R^2	Pr (>F)
**Water**	Banyuls vs Sète	0.1262	0.0138 (*)
	Banyuls vs Thau	0.25348	0.0012 (**)
	Sète vs Thau	0.11507	0.0213 (*)

(C) Statistics of the beta diversity using the DEseq2 normalization and Unifrac distance matrix depending Site variable within Sediment samples.

Adonis test on multiple variables
Permutation: free
Number of permutations: 9999

		R^2	Pr (>F)
**Sediment**	Banyuls vs Sète	0.11439	0.0044 (**)
	Banyuls vs Thau	0.27517	0.0001 (***)
	Sète vs Thau	0.25265	0.0001 (***)

Table S6

(A) Statistics of the alpha diversity using the Chao1, Shannon and InvSimpson diversity indexes on Strain variable.

ANOVA on Chao1, Shannon and InvSimpson diversity index

		Chao1		Shannon		InvSimpson	
		F value	Pr (>F)	F value	Pr (>F)	F value	Pr (>F)
**Strain**	SBS363 vs A01	4.964	0.0313 (*)	1.063	0.308	2.056	0.159
	SBS363 vs LGP32	0.275	0.602953	3.293	0.0769	3.279	0.0775
	SBS363 vs J2-9	0.237	0.62847	0.176	0.677	0.036	0.850
	A01 vs LGP32	6.951	0.011692 (*)	7.632	0.00847 (**)	9.680	0.00334 (**)
	A01 vs J2-9	2.646	0.11076	1.523	0.224	1.183	0.282
	LGP32 vs J2-9	0.792	0.378344	1.652	0.205	2.397	0.129

(B) Statistics of the beta diversity using the DEseq2 normalization method and Unifrac distance matrix depending on Strain variable.

Adonis test on multiple variables
Permutation: free
Number of permutations: 9999

		R^2	Pr (>F)
**Strain**	SBS363 vs A01	0.01926	0.5398
	SBS363 vs LGP32	0.02299	0.4333
	SBS363 vs J2-9	0.02015	0.481
	A01 vs LGP32	0.02948	0.22
	A01 vs J2-9	0.03109	0.1376
	LGP32 vs J2-9	0.0178	0.61
